# Clinical Holistic Medicine: Holistic Adolescent Medicine

**DOI:** 10.1100/tsw.2004.112

**Published:** 2004-08-04

**Authors:** Søren Ventegodt, Mohammed Morad, Joseph Press, Joav Merrick, Daniel T.L. Shek

**Affiliations:** ^1^ The Quality of Life Research Center, Teglgårdstræde 4-8, DK-1452 Copenhagen K, Denmark; ^2^ Division of Community Health, Ben Gurion University, Beer-Sheva, Israel; ^3^ Division of Pediatrics, Soroka University Medical Center, Ben Gurion University, Beer-Sheva, Israel; ^4^ 4National Institute of Child Health and Human Development, Office of the Medical Director, Division for Mental Retardation, Ministry of Social Affairs, Jerusalem and Zusman Child Development Center, Division of Pediatrics, Ben Gurion University, Beer-Sheva, Israel; ^5^ Department of Social Work, Chinese University of Hong Kong, Shatin, Hong Kong

**Keywords:** quality of life, QOL, philosophy, human development, holistic medicine, public health, holistic health, holistic process theory, life mission theory, group therapy, adolescent medicine, Denmark

## Abstract

The holistic medical approach seems to be efficient and can also be used in adolescent medicine. Supporting the teenager to grow and develop is extremely important in order to prevent many of the problems they can carry into adulthood. The simple consciousness-based, holistic medicine — giving love, winning trust, giving holding, and getting permission to help the patient feel, understand, and let go of negative beliefs — is easy for the physician interested in this kind of practice and it requires little previous training for the physician to be able to care for his/her patient. A deeper insight into the principles of holistic treatment and a thorough understanding of our fellow human beings are making it work even better. Holistic medicine is not a miracle cure, but rather a means by which the empathic physician can support the patient in improving his/her future life in respect to quality of life, health, and functional capacity — through coaching the patient to work on him/herself in a hard and disciplined manner. When the patient is young, this work is so much easier. During our lifetime, we have several emotional traumas arranged in the subconscious mind with the smallest at the top, and it is normal for the person to work on a large number of traumatic events that have been processed to varying degrees. Some traumas have been acknowledged, some are still being explored by the person, and yet others are still preconscious, which can be seen for example in the form of muscle tension. Sometimes the young dysfunctional patient carries severe traumas of a violent or sexual nature, but the physician skilled in the holistic medical toolbox can help the patient on his/her way to an excellent quality of life, full self-expression, a love and sex life, and a realization of his/her talents — all that a young patient is typically dreaming about. Biomedicine is not necessary or even recommended when the physical or mental symptoms are caused by disturbances in the personal development that can be corrected with love and understanding. If possible, biomedicine must be avoided, even if this means suffering for the young person, who needs to confront the tough realities of life in order to grow into an able and sound adult.

## INTRODUCTION

Adolescent medicine is becoming a new subspecialty[1,2,3], but it will always be an integral part of general practice and pediatric medicine. With teenagers, preventive medicine is very important because many problems can be solved and prevented in this age by the physician teaching what needs to be known about sex, lifestyle, and philosophy of life in order to avoid the many problems haunting young people today: pregnancy and contraception[4], HIV and AIDS[5,6], substance use and abuse[7,8,9], ethics, law, sports[10], violence[11], prostitution and victimization[12]. In adolescent medicine, psychosomatics is very important and related to the 20–30% or so of the teenagers suffering from either chronic pains[13], psychiatric disturbances[14,15,16], eating disturbances[17,18,19], vulvodynia or other gynecological problems[20,21,22].

Most of the problems can be seen as disturbances in the psychosocial and sexual development, often with patterns going back to childhood[23]. Often it is some severe unsolved traumas that have blocked the personal development and then later on present with symptoms. The incidence of sexual abuse in eating disorder patients is an example; 50% of both our anorectic and bulimic patients reported a history of sexual abuse, while only 28% of a nonanorexic, nonbulimic control population reported similar problems[24], so we recommend that sexual issues be addressed early in the treatment of adolescents with eating disorders.

Let us examine what consciousness-based medicine can offer to the teenager within a general and traditionally nonmedical field, namely personal development. That is not one of the strengths of biomedicine, but consciousness-based medicine is obviously relevant. Regardless of the motive for visiting a physician, consciousness-based medicine is primarily a means of help for personal development. It will improve your general condition over a period, often months, and increase the general energy level in the process, mobilize hidden resources and self-healing powers. Accordingly, consciousness-based medicine is very useful for rectifying chronic disorders and existential problems, so common in the teenage years. Sexual problems are very common and important for the adolescent to solve and here the holistic physician can be of great help[25,26,27]. Even with very serious issues, like sexual abuse, the consciousness-based medicine will enable the teenager not to deny their sexuality, punish themselves or others, and problems like guilt, depression, repressed anger, low self-esteem, social isolation, and inadequacy are often alleviated. Because of the vulnerability of young people, the “holding and love” of the holistic physician is extremely important.

As with adults, it is often relevant and necessary to supplement consciousness-based medicine with biomedicine in the acute phase of a disease of a child and an adolescent. This can be done with hardly any problems, since biomedicine and quality of life (QOL) address the problem from two independent angles. There is a risk, however, that biomedicine softens the symptoms so much that, from a consciousness-oriented medical view, it removes the incentive and important guidemarks in the patient’s own efforts to improve his life and state of health. Pain is meaningful in holistic medicine, because the body and mind use pain to tell the patient something important about the imbalances of life. The pain is a primary source of learning.

The biological understanding of the correlation between quality of life and physical and mental illness is still incomplete. We believe that the explanation for the great coherence lies in the information systems of the body[28,29], as our experiences and consciousness are continuous with the collective field of information applied by the cells to communicate. In the healthy body, this information system ensures that all cells know exactly what to do in their particular location. Disease seems to occur when a person in a traumatic situation unconsciously stores emotional blockages in tissue and organs, which subsequently disturb the information flow between the cells and thus the functions of the cells and tissue. That we believe can cause physical and mental illness.

To heal the body and mind, the cells in the body and brain must be made to communicate freely once again. This will happen when the blockage is released as explained by the holistic process theory. Healing occurs as the person lets go of the decisions that originally led to the suppression of the painful feelings.

As the person lets go of many of the negative and responsibility-destructive decisions, life perspective will gradually and naturally turn positive. The person becomes happier and more attentive, more responsible for his/her own life, with the body and mind starting to heal. A patient who is prepared to let go of his/her entire life-denying perspective, and does so effectively and purposefully, may experience healing in a surprisingly short time of a physical or mental illness, sometimes even cancer or heart failure. That is what we call spontaneous remissions. If the patient strongly resists the process, this may take years or not occur at all.

## CLINICAL HOLISTIC MEDICINE IS BASED ON THE LIFE MISSION THEORY AND THE HOLISTIC PROCESS THEORY OF HEALING

The life mission theory[23,30,31,32,33,34] concludes that everybody has a purpose of life or huge talent. Happiness comes from living this purpose and succeeding in expressing the core talent in your life. To do this, it is important to develop into what is known as the natural condition, a condition where you know yourself and use all efforts to achieve what is most important to you, like good relations [35]. The holistic process theory of healing[36,37,38,39] and the related quality of life theories[40,41,42] state that the return to the natural state of being is possible whenever the person gets the resources needed for existential healing. The resources needed are in the dimensions awareness, respect, care, acknowledgment, and acceptance with support and processing in the dimensions feeling, understanding, and letting go of negative attitudes and beliefs. The preconditions for holistic healing to take place are trust and the intention for the healing to take place. Existential healing is not a local healing of any tissue, but a healing of the wholeness of the person, making him/her much more resourceful, loving, and knowledgeable of him/herself and his/her own needs and wishes. In letting go of negative attitudes and beliefs, the person returns to a more responsible existential position and an improved quality of life. The philosophical change of the person healing is often a change towards preferring difficult problems and challenges, instead of avoiding difficulties in life[43,44,45,46,47,48,49,50]. The person who becomes happier and more resourceful often also becomes more healthy, more talented, and more able to function[51,52,53]. The concepts underlying the life mission theory and the holistic process theory of healing are consistent with the emphasis of life meaning in logotherapy, as proposed by the late Austrian Victor Frankl (1905–1997) and the notion of transcendence embedded in existential therapies.

## QUALITY OF LIFE FOR ADOLESCENTS

Adolescents will visit their physician with many health-related issues, like functional and sexual symptoms, acne, obesity and eating disorders, depression, anxiety, and communication problems with adults and other adolescents. Surveys of adolescents have shown that about 10% have chronic illness and only about 10–15% of all surveyed thought they were healthy[54]. It is hard to be young, and many youths do not have the best of terms. Their childhood did not provide them with the good self-image, the belief in life and in the world, which is required in order to attain a good life.

Basically, there are two problems. The problem is either self-confidence — the young person may feel inadequate at school or things are not working out with friends or girl/boyfriend, or there is a problem of self-esteem — the young person feels inferior, hopeless, useless, and worthless. Problems of selfconfidence and self-esteem are manifested in many ways. For example, suicidal thoughts, which often occur when self-esteem turns negative and the person regards him/herself as being in the way and an inconvenience. “I don’t deserve to live, I wish I was dead.” Another example is drug abuse. “I am only in control when I am high.” Or school fatigue: “I’m no good at reading.” Other cases are about conflicts with parents or other adults. “They don’t understand me.”

> **Female, aged 14 years, with severe pain in right wrist:** Persistent severe pain in right wrist in particular. Examinations: X-ray showed nothing abnormal. Ligaments seemed slightly loose. Slightly grating joints. Should be loosened up. Appears very tense in the upper arms, shoulders, and neck. This tension may explain the complaints of wrist pain. We talked about the patient being a nice girl, who becomes tense in an attempt to keep herself in place and do what is expected of her. EXERCISE: Be more free, bold, and cheerful. Can return in a month.

Young people do not have a rigid personality and are therefore able to adapt quickly when they realize a connection. The hand hurts. Why does it hurt? Well, because she is tense. Why is she tense? Because she tries to be nice and correct, instead of being herself, bold, cheerful, and free. A single conversation makes her realize the connection and hopefully solves the problem.

## SELF-CONFIDENCE AND SELF-ESTEEM CAN BE DEVELOPED

Often, it is possible to help young people considerably with their problems in a short time if we focus on their personal development. Young people have remarkable potential for improving their quality of life, because their egos are not yet so firmly developed as to prevent a change in life philosophy. If the procedure can be agreed on, the young person can work intensely on the problems when they are acute, and subsequently continue working, less intensely, for a long time to keep the development going. The process should be agreed on, for example, from three to ten conversation sessions.

> **Female, aged 15 years, with a life crisis:** First QOL conversation: Has not attended school for 1 year, she just lies on her bed sleeping every day. Lives with a friend. Her mother threw her out and says that she sold her bed. She did well in primary and lower-secondary school, scored top grades, but the problems began when her mother moved her to a private school. On bad terms with her mother since then. Would like to return to her old school, but there are no vacancies in her old class. She feels bad about herself. Her stomach is too fat, her ears need to be moved, calf muscles too big, bunions on both feet, apple-cheeked, pimples, hair has split ends. Good things: small feet, feminine hands, she is smart and small, and her eyes are pretty. Examination: She is growing up too fast, she should return to school and teenage life, if possible. The problem is her pride, which prevents her from adapting to a life as a child and pupil. Diagnosis: /life crisis/. EXERCISE: practice humility by not reacting with pride whenever she is tempted to do so.
>
> Second QOL conversation: Since last session: Great improvement. She is now prepared to return to school, pass her exams, complete the $10^{th}$ grade and be a young student again. Her direction in life has straightened up, she is happy again and on the right track. She has thought about our conversation – wants to be both child and adult. Has a boyfriend. Has acquired lots of energy and drive. For example, she gets up early in the morning and tidies up. She will get the books for her exam tomorrow, and should make a study plan. She has a woman friend who helps her. Should find a quiet place to study, e.g., the library. Has sufficient discipline to succeed. Should return in 2 weeks, so that we make sure that everything is proceeding well.

Third QOL conversation: Has had three exams, they went well. Is now awaiting oral exam. Looks very well. And is happy. Will be starting in 10th grade after the summer holidays. Her friend has been a great help. The patient says that the conversations helped her attain a different outlook on life. Should return in 6 months for a “check-up”.

This young girl’s life had gone completely off track and she realized that herself. Her problem was her lack of respect for adults. Nevertheless, she chose to respect the physician, she accepted this help willingly, and took responsibility for her life. She agreed to practice humility instead of pride and set her life straight. Things could have gone very wrong for her, as she was very far out. She did very well in swallowing her pride, which concealed her lack of self-esteem.

**Female, aged 14 years, with psychosomatic abdominal pain:** First visit: Complains of abdominal pain so severe that sometimes she can hardly move. This worsens at the time of menstruation. A couple of weeks ago, the abdominal pain lasted a whole week. Often, it also hurts in the morning before school. Given appointment for assessment.

Second visit: Chronic abdominal pain with almost daily episodes, since her parents’ divorce 2 years ago. Pain most intense in the afternoon and again in the evening. Pain in the morning, before school almost daily. Sometimes so intense that she screams. Has to undo her trousers. Now also diarrhea for 2 weeks, 4-5-6 bowel movements daily. No blood or purulent matter, but mucus in the feces. Pain corresponding to both solar plexus and above the symphysis. Gynecological: Does not know whether she has vaginal discharge. 28/4-5, next menstruation in 7 days. Occasional abdominal pain during sexual intercourse, which then has to be interrupted. Pelvic examination referred to a gynecologist. Blood samples drawn. Possibly assessment for suspected ulcerative colitis/irritable bowel syndrome at hospital. Decision awaits blood test results. Interview with the mother, who is unhappy about the relationship with the boyfriend, whom she sees too much. The patient is not allowed to attend the same school as him. The patient feels pressured by her mother, who wants her to concentrate more on school.

The unspoken tensions are obvious. Her mother does not accept her daughter and her life, her liberation, her boyfriend, or her sexuality. The mother transfers her lack of acceptance of herself and all the problems in her own life straight to the daughter, who has not yet become so independent that she can avoid suffering. Our task is to make the young person focus on herself and her own needs and be 100% selfish and in that way break her mother’s control of her. As the daughter is the patient, we must be totally on her side. The odd thing is that they can both be entirely selfish and yet generously good to each other at the same time. We become frustrated, because it is in a deadlock, as the mother is not ready to let go of her daughter. It is as if she has no other content in her life than the relationship with her daughter.

## WORKING WITH A TEENAGER WITH A MENTAL DISORDER

If one of our young patients is diagnosed as mentally ill, but poses no danger to self or others, we may offer the patient conversational therapy to improve his/her quality of life. If the young person suffers severe mental problems, we usually suggest cooperation with one of the skilled psychiatrists we work with, who is psychodynamically oriented. In our opinion, a mental disorder *per se* is not a sufficient cause to commence medical treatment. We believe that the patient needs the personal suffering to be genuinely motivated for his/her personal development, which in the long term is to free the patient from his illness. The recovery literature shows several kinds of recovery from schizophrenia[55], the most interesting being full recovery happening in one study in 13.7% of the patients after 5 years[56] and in about 25% of the patients in the long run[57,58] in the western countries and quite surprisingly much more often in the third world[59]. It is important to cooperate with this spontaneous recovery process.

In our experience, there is no obstacle to therapeutic work according to the principles described later in patients who are psychotic and have problems with reality testing. Therapy may help the patient face his/her own inner reality and restore the relationship with the surrounding world on that basis. Generally, we use as little sedative and antipsychotic medication as possible to avoid delaying the development process and thereby letting the patient suffer from the mental disease for longer than necessary. Naturally, the amount of drugs should be sufficient to prevent the patient from being hospitalized, because hospitalization in itself can have negative effects on perception of self and also to avoid irreparable damage to the patient's reputation and social network, e.g., during a manic episode. However, young people often experience mental problems of a milder nature, which can easily be cured if the patient "is met" with care and understanding and instructed in appropriate exercises and tasks.

> **Female, aged 18 years, with pain in lower limbs, concealing anxiety and grief:** First visit: Severe pain in lower limbs for 2 or 3 months, episodes twice a week. The legs go numb, also many cramps. Examination: very tense in both thighs and buttocks, tension corresponding to the trigger points in the lower half of the body, which is massaged, but no problems with trigger points in the upper half. Shy, somewhat reserved, has problems, also with boys, "who are no better than a heap of crap" and with love. New appointment for conversation. History of pelvic discomfort, including vaginal discharge, itching, and pain treated with Zithromax [azithromycin] due to Chlamydia infection.
>
> Second visit: We talk about all the feelings buried in the body. Anxiety attacks, which she suffered from some years ago and which still appear when she is going to sleep so that she is unable to breathe and feels confined in her body and just has to get out. Sadness because she is lonely when she is alone, depression about a year ago, now the feeling of being "arid" and numb. Love is difficult, we may talk about that the next time. EXERCISE: Accept and accommodate your feelings: dwell on your feelings when they are bad, set the egg-timer at 15 minutes and dwell on your anxiety for that long; when it goes off, write the feeling down on paper and bring it here so that we may talk about it.

It takes skill to turn the attention away from the legs and the hurt to make her concentrate on the important issues in life, which are nothing like what they are supposed to be. Holistic medicine is about working with one's health and existence — the soul. The moment the patient realizes that recovery is about life perspective, then much can be learned from the symptoms or disease and the scene changes from a meaningless hell to a constructive struggle for understanding and survival. Our patients have sometimes wanted a new perspective of their lives for a long time. At the clinic, we may suddenly feel like giving the patient an exercise. Exercises are processes — often quite complex processes — that the patient must undergo at home. In our experience, the exercise will only work if it receives an emotional GO from the patient, in the specific situation when it was given. The inspiration to give the patient the exercise is our intuitive reaction to the patient's breakthrough to a new, constructive life perspective. Sometimes, the shift in perspective at the clinic may take just a few minutes and it occurs when a motivated patient receives the requisite support.

> **Female, aged 19 years, with existential problems and loneliness:** First QOL conversation: Existential problems manifested as back pain, reduced sexual desire, and chronic loneliness. She has suffered tension in the thoracic and cervical spine for years, has seen a psychologist without feeling that she was helped. Suffers from "breakfast-table grumpiness", sadness, sullenness, and anger released in uncontrollable onslaughts, when she is provoked. She is chronically surly and generally suppresses her feelings, also her

> sexual drive and enjoyment of life. Socially: She does not have a boyfriend and it seems few close friends. Apparently, she generally distrusts other people and finds it difficult to open up, not least to a male (SV). Examination: Tension corresponding to the long thoracic muscles of the back, there is slight, but very distinct locking in the back of the neck (muscle tension of an older date). She does not breathe down in the abdomen and pelvis, where there are many tensions and old feelings — anger and also coldness. Is prepared to work on herself. PLAN: SV week 6 + week 12 + as required, holistic body therapy × 6 every 2 weeks, starting next week, Rosen sessions × 6 every 2 weeks, starting next week (rhythm and extent to be determined by Rosen practitioner)[60].
>
> Second QOL conversation: She has had three to four body therapy sessions and one Rosen session. Feels a little happier. Other people can tell that she is by her appearance. Three weeks ago she found a boyfriend and all in all she is doing fine. She is still somewhat surly and is often cross with her boyfriend. She always got along better with boys than with girls, she says, and appears somewhat masculine. We discuss that. Clearly, she has to deal with some surliness and then some anger before being able to express herself in the way she wants to. At present, she understands very little of what is going on. I recommend a male Rosen practitioner.
>
> Third QOL conversation: She has now had four or five Rosen sessions, feels discomfort when touched on the stomach: go away! I sense something dark and depressive in the patient. We work on that: "Daddy, you are hurting me." The patient feels pain, feel rigid, sweaty hands, shivers, and flushes. Suddenly the patient is 3 or 4 years old, and it looks as if her father rejects her love. On the couch, we work on the blockages on her chest and abdomen. The patient is clinging to the "wall", which slowly becomes thinner. I get an image of a jeep stuck in the mud, more and more ready to take off. To continue the Rosen sessions, new appointment with me in 1 month.

This story shows that existential conditions such as loneliness and isolation can be changed through targeted work with the problems. The young woman doing it quickly makes progress. It should be noted that already she goes out and breaks her social isolation, finding the boyfriend that she has been lacking.

## DISCUSSION

Biomedicine is often not recommended for young people, as their health problems often are symptoms of developmental problems that they have to solve to be able adults. Biomedicine can often take away the pain and the symptoms, but by doing so the adolescent is deprived of his/her motivation for reflecting and learning, alienated to body and/or mind and the processes of healing itself; in the long run this can be a very high price to pay for a momentarily pain relief. Excessive preoccupation with biochemical treatment would also cultivate "sick roles" in adolescents and it neglects the "deeper" issues that are encountered by the adolescent concerned.

Our style of holistic medicine is not pain free; it actually hurts a lot. As the patient moves forward and upwards on the scale of self-esteem, quality of life, and personal resources in order to assume responsibility, access, and integrate past traumas, the hurt is deep, painful, and existential. In our experience, the patient's life increasingly resembles a roller coaster, as the holistic therapy moves towards its conclusion. Strong emotions, both positive and negative, both related to the outer world and not related at all, are all good signs of progress in the therapy. Strong, intense emotions simply mean that the patient is becoming alive once again.

Perhaps the true, natural life is an alternating state, with severe pain being replaced by great and profound happiness. Perhaps the therapy does not make the patient free from pain, but rather alive and capable of experiencing life for better and for worse. Consciousness-based medicine aims at moving the problems from the body and mind to existence, but life does not become the romantic dream we sometimes see depicted in the movies or books. Rather, life becomes an existence in an enchanted jungle, filled with wonderful and dangerous things.

It is very important to give the young patient the right expectations to the therapy and the outcome, namely an ability to live a conscious and responsible life, an ability to love again, and thus also an ability to feel once again the pain of rejection and the fear of vulnerability, but to be able to put in its right place. To be alive is not to be safe or pain free, because life is pain and danger, but it is important to find the joy of life and take responsibility for your choices. This attitude can make life worth living.

## CONCLUSIONS

The holistic medical toolbox seems to be highly efficient and can be used in adolescent medicine. Supporting the teenager to grow and develop seems to be extremely important in order to prevent many of the problems that we see in adults, where developmental problems were not solved. Of course, more research is needed to further strengthen the empirical bases of holistic medicine in an era that stresses the importance of evidence-based practice.

The simple consciousness-based, holistic medicine — giving love, winning the patients trust, giving holding, and getting permission to help the patient feel, understand, and let go of negative beliefs — is easy for the physician interested in this kind of practice. It requires little previous training for the physician who is able “to love and care” for his/her patient. A deeper insight into the principles of holistic treatment and a thorough understanding of our fellow human beings are making it work even better.

Holistic medicine is not a miracle cure, but rather a means by which the empathic physician can support the patient in improving his/her future life in regard to quality of life, health, and functional capacity. This can be done by coaching the patient to work on him/herself in a hard and disciplined manner, which is so much easier when the patient is young. In terms of training, it means that the simple acquisition of medical knowledge is inadequate to train an empathic physician. As such, medical schools should review the related curricula to see whether the physicians trained identify with the philosophies and principles of holistic medicine.

As we all experience many emotional traumas during a lifespan, and as the traumas are arranged in the subconscious mind so that the smallest lie at the top, it is normal for the patient to work on a large number of traumatic events that have been processed to varying degrees. Some traumas have been acknowledged, others are still being explored by the patient, and yet others are still preconscious. As the “internal waste bin” is emptied, the patient’s general mood and well being will gradually improve, but the traumas that are uncovered date further and further back and are increasingly negative. Sometimes the young dysfunctional patient carries severe traumas of a violent or sexual nature and the physician skilled in the holistic medical toolbox can often help the patient on his/her way to improve quality of life, self-expression, love and sex life, and to realize big hidden talents — all that a young patient is typically dreaming about.

Biomedicine is not necessary or even recommended when the physical or mental symptoms are caused by disturbances in the teenagers’ personal development that can be corrected with love and understanding[23]. Biomedicine must be avoided if at all possible, even if this means suffering for the young person, who needs to confront the tough realities of life to grow into an able and sound adult.

## ACKNOWLEDGMENTS

This study was supported by grants from IMK Almene Fond. The quality of life research was approved by the Copenhagen Scientific Ethical Committee under number (KF)V.100.2123/91.

## BIOSKETCHES

**Søren Ventegodt, MD,** is the Director of the Quality of Life Research Center in Copenhagen, Denmark. He is also responsible for a Research Clinic for Holistic Medicine in Copenhagen and is a popular speaker throughout Scandinavia. He has published numerous scientific or popular articles and a number of books on holistic medicine, quality of life, and quality of working life. His most important scientific contributions are the comprehensive SEQOL questionnaire, the very short QoL5 questionnaire, the integrated QOL theory, the holistic process theory, the life mission theory, and the Danish Quality of Life Research Survey, 1991–94 in cooperation with the University Hospital of Copenhagen and the late pediatric professor Bengt Zachau-Christiansen. E-mail: ventegodt@livskvalitet.org. Website: http://www.livskvalitet.org

**Mohammed Morad, MD**, is Specialist in Family Medicine, Lecturer in Family Medicine at the National Institute of Child Health and Human Development, Division of Community Health, Ben Gurion University of the Negev and the Medical Director of a large area clinic in the city of Beer-Sheva. He has publications on Bedouin health, health aspects, spiritual health, and aging in persons with intellectual disability, and is a presenter on topics such as health policy and services for the disadvantaged at national and international conferences. E-mail: morad62@barak-online.net

**Joseph Press, MD**, is Professor of Pediatrics with interest in emergency medicine and rheumatology. He serves as the Director of the Pediatric Emergency Department and Chairman of the Pediatric Division, Soroka University Medical Center, Faculty of Health Sciences, Ben Gurion University of the Negev and the prime mover behind the construction of the Saban Children's Medical Center at Soroka. E-mail: press@bgumail.bgu.ac.il

**Joav Merrick, MD, DMSc,** is Professor of Child Health and Human Development affiliated with the Zusman Child Development Center, Division of Pediatrics and Community Health at the Ben Gurion University, Beer-Sheva, Israel; the Medical Director of the Division for Mental Retardation, Ministry of Social Affairs, Jerusalem; and the Founder and Director of the National Institute of Child Health and Human Development. He has numerous publications in the field of child and human development, rehabilitation, intellectual disability, disability, health, welfare, abuse, advocacy, quality of life, and prevention. Dr. Merrick received the Peter Sabroe Child Award for outstanding work on behalf of Danish Children in 1985 and the International LEGO-Prize ("The Children's Nobel Prize") for an extraordinary contribution towards improvement in child welfare and well being in 1987. E-mail: jmerrick@internet-zahav.net. Website: www.nichd-israel.com

**Daniel T.L. Shek, PhD, FHKPs.S, BBS, JP**, is Professor of Social Work at the Department of Social Work, the Chinese University of Hong Kong. He is on the editorial board of several scientific journals and has published extensively in international journals on social work, Chinese and English language, mental health, well being, quality of life, adolescence, disadvantaged families, and drug abuse. E-Mail: DANIELSHEK@CUHK.EDU.HK. Website: http://www.cuhk.edu.hk/swk
